# Heartworm disease in domestic dogs in Estonia: indication of local circulation of the zoonotic parasite *Dirofilaria immitis* farther north than previously reported

**DOI:** 10.1186/s13071-024-06217-5

**Published:** 2024-03-12

**Authors:** Maare Mõttus, Paul F. Mõtsküla, Pikka Jokelainen

**Affiliations:** 1 Estonian University of Life Sciences, Tartu, Estonia; 2 Infectious Disease Preparedness, Statens Serum Institut, Copenhagen, Denmark

**Keywords:** *Dirofilaria immitis*, Europe, Emerging infection, Heartworm, Zoonosis

## Abstract

**Background:**

The mosquito-borne zoonotic parasite *Dirofilaria immitis* continues to spread northwards in Europe. This parasite can cause potentially life-threatening heartworm disease in dogs and pulmonary dirofilariasis in humans and is, therefore, a major health concern in both the veterinary medicine and human medical fields. This is the first report of *D. immitis* infections and heartworm disease in the Baltic country Estonia.

**Methods:**

Data on canine *D. immitis* infections and heartworm disease were collected from the electronic patient records database of the Small Animal Clinic of Estonian University of Life Sciences, the only university clinic in Estonia. The patient records of dogs with confirmed diagnosis of *D. immitis* infection or heartworm disease were reviewed and summarised.

**Results:**

Six dogs had been diagnosed with confirmed *D. immitis* infection or heartworm disease at the university clinic in 2021–2022. The confirmed diagnoses had been reached following international guidelines, based on a combination of different tests. Molecular confirmation of the parasite species had not been performed. Two of the dogs had been imported while four had no travel history outside of the country.

**Conclusions:**

Four of the dogs with a confirmed *D. immitis* infection or heartworm disease had no history of being imported or travelling outside of the country, indicating autochthonous infections and, consequently, local circulation of the parasite in Estonia. These findings represent the new northernmost autochthonous cases of *D. immitis* infection and canine heartworm disease reported in the European Union.

**Graphical Abstract:**

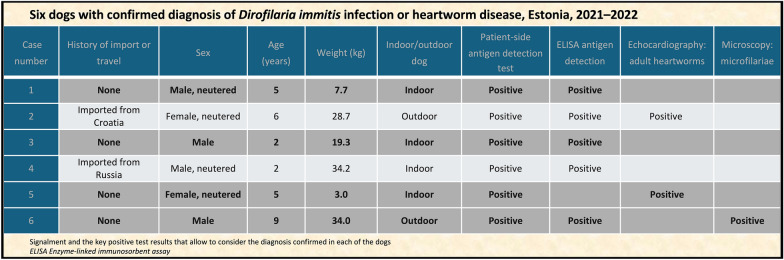

## Background

*Dirofilaria immitis* (Leidy, 1856) is the causal agent of heartworm disease in domestic and wild carnivores [1]. The disease is well known as affecting domestic dogs, while the infection has been reported in > 30 mammalian species [2–4]. *Dirofilaria immitis* is zoonotic and can infect humans in whom it causes, for example, pulmonary dirofilariasis [3, 5, 6].

The clinical signs of canine heartworm disease can vary from mild and nonspecific to life-threatening. Typically reported clinical signs include weakness, exercise intolerance, lethargy, depression, dehydration, cough, dyspnoea, cachexia, ascites, pale mucous membranes and exertional syncope [3, 7]. The most common cause of death in dogs with severe heartworm disease is right-sided heart failure [8, 9].

*Dirofilaria immitis* has a wide distribution in the world, and its endemic areas have been expanding during the last decade [10, 11]. It is a vector-borne pathogen, with numerous mosquito species, including *Aedes* spp., *Anopheles* spp. and *Culex* spp., reported as vectors [12–14]. Almost 80% of the mosquito species described in Estonia belong to these genera [15].

Numerous studies on *D. immitis* have been conducted in endemic areas in Europe and North America [11, 16–18], while few available studies originate from northeastern Europe. Autochthonous cases of *D*. *immitis* infection have been described in Russia [19], Poland [20] and Belarus [21], while a recent review found no published reports of autochthonous *D. immitis* infection in dogs from Denmark, Finland, Iceland or Norway [22]. *Dirofilaria* spp. were reported in several dogs in Sweden, but species-level information was not provided [22, 23].

In addition to *D. immitis*, another zoonotic parasite of the same genus, *Dirofilaria repens*, with largely similar requirements as *D. immitis* for vectors, hosts and environmental conditions [3, 24], has been detected as an imported species in northeastern European countries as well as reported to have spread northwards [22, 25–27]. *Dirofilaria repens* is already established and considered to be endemic in the Baltic countries, including Estonia [28–30]. Thus, the emergence of *D. immitis* in Estonia has been anticipated. The northernmost autochthonous *D. repens* infection reported in the European Union so far was in Finland, which is located north of Estonia [26].

Previously, a questionnaire-based study was conducted among veterinarians in the Baltic countries (Estonia, Latvia and Lithuania) and the Nordic countries (Denmark, Finland, Iceland, Norway and Sweden) to estimate the proportion of veterinarians that had seen cases of canine babesiosis, dogs with *D. immitis* infection or dogs with *D. repens* infection during 2016 [31]. A total of 122 veterinarians participated in the study, among whom 18 (15%) reported having seen at least one dog with *D. immitis* infection and 11 (9%) reported having seen at least one dog with *D. repens* infection in 2016.

The first internationally published description of *D. immitis* infection in the Baltic countries was in an imported dog, in 2019, in Lithuania [32]. Literature searches did not identify any available publications on *D. immitis* from Estonia. However, canine cases have been seen in recent years (unpublished observations).

The aim of this study was to summarise confirmed diagnoses of *D. immitis* infection or heartworm disease in dogs from Estonia.

## Methods

The electronic patient records of the database (Provet Cloud, Espoo, Finland) of the only university animal clinic in Estonia, the Small Animal Clinic of the Estonian University of Life Sciences, were searched for canine patients diagnosed with *D. immitis* infection. The search was done targeting the final diagnosis and by using “dirofilariosis” and “*Dirofilaria* spp. infection” as filters, with no time limitation. All results of the search were reviewed, and canine patients with confirmed *D. immitis* infection or heartworm disease were included in the case series. The last search was conducted on 30 June 2023. The full patient records of the included dogs were extracted from the system and reviewed by two authors (MM and PFM). This study was observational and did not affect the clinical management of the cases. In the framework of this retrospective study, the authors had no direct contact to the dogs or the owners of the dogs, and no means of verifying the data in the patient records.

## Results

### Case series

Six dogs with *D. immitis* infection as their final diagnosis were included in the case series.

#### Case 1

Case 1 was a 5-year-old, small (7.7 kg), neutered male crossbreed indoor dog that had been adopted from an animal shelter in Estonia 3 months prior to the diagnosis. The previous medical history of the dog was unknown. The dog was brought to the clinic for investigation of lameness. On physical examination, a mild right-sided murmur was noted, and the patient was referred to a veterinary cardiologist. A patient-side antigen detection test (SNAP® 4Dx®; IDEXX, Westbrook, Maine, USA) was positive for heartworm antigen, and serology by enzyme-linked immunosorbent assay (ELISA) to detect *D. immitis* antigen (Dirofilaria—Antigen; LABOKLIN, Bad Kissingen, Germany) was positive. Echocardiography revealed mild tricuspid valve regurgitation, the velocity of which (TR Vmax 2.10 m/s) was not suggestive of pulmonary arterial hypertension; the remainder of the echocardiographic examination was unremarkable. The treatment was planned and conducted according to the recommended management protocol of the American Heartworm Society (AHS) [33] and the protocol included strict restriction of exercise and treatment with doxycycline, selamectin, prednisolone and melarsomine dihydrochloride.

#### Case 2

Case 2 was a 6-year-old, large (28.7 kg), neutered female crossbreed outdoor dog. One month before the diagnosis, the dog had been adopted from an animal shelter, and it had been imported from Croatia a few weeks earlier. The dog was brought to the clinic for investigation of exercise intolerance, cough and inappetence. The physical examination was largely unremarkable. The patient-side antigen detection test was positive for heartworm antigen, the ELISA was positive for *D. immitis* antigen, while the Knott test (Knott test; LABOKLIN, Bad Kissingen, Germany) and real-time PCR (microfilaria-PCR; LABOKLIN) were negative. Examination of thoracic radiographs revealed severe dilation of the pulmonary arteries and a mild diffuse interstitial lung pattern. On echocardiography, filamentous structures consistent with adult parasites were clearly visible in the pulmonary arteries. The treatment protocol included strict restriction of exercise and treatment with milbemycin oxime/praziquantel, doxycycline and melarsomine dihydrochloride.

#### Case 3

Case 3 was a 2-year-old, medium-sized (19.3 kg), male indoor dog. The dog was referred to a veterinary cardiologist for further investigation of cough and leukocytosis; the owner also reported that the dog had vomiting and diarrhoea. The patient-side antigen detection test was positive for heartworm antigen, and the ELISA was positive for *D. immitis* antigen. The echocardiographic examination was unremarkable. The treatment protocol included restriction of exercise and treatment with milbemycin oxime/afoxolaner, doxycycline and melarsomine dihydrochloride.

#### Case 4

Case 4 was a 2-year-old, large (34.2 kg), neutered male crossbreed mostly indoor dog. The dog had been adopted 6 months before the diagnosis from a dog shelter and had been originally imported from Russia. The first visit to a local veterinary clinic was a few days after the adoption due to a post-surgical infection. The owner reported that the dog had laboured breathing pattern at rest. Dermatological and dental problems were also noted. Before a planned dental procedure, the local veterinarian performed the patient-side antigen detection test, which was positive for heartworm antigen. The dog was referred to a veterinary cardiologist. The ELISA for *D. immitis* antigen was positive, while the Knott test and the real-time PCR were negative. The echocardiographic examination was unremarkable. The treatment protocol included strict restriction of exercise and treatment with milbemycin oxime/afoxolaner, doxycycline and melarsomine dihydrochloride.

#### Case 5

Case 5 was a 5-year-old, small (3.0 kg), neutered female crossbreed indoor dog. The dog was referred to a veterinary cardiologist for suspected cardiomegaly, heart murmur, cough and pyrexia. To rule out *Angiostrongylus vasorum* infection [34, 35], an antigen detection test (Angio Detect Test®; IDEXX) was performed, and the result was negative. The patient-side antigen detection test was positive for heartworm antigen, and echocardiography revealed linear structures in the right side of the heart and in the pulmonary arteries. There was also echocardiographic evidence of mild pulmonary hypertension. The treatment protocol included treatment with an unspecified macrocyclic lactone, doxycycline, prednisolone and melarsomine dihydrochloride, and cage rest.

#### Case 6

Case 6 was a 9-year-old, large (34.0 kg), male crossbreed outdoor dog. The dog was brought to the clinic for investigation of anorexia, vomiting and unwillingness to move. The physical examination revealed no significant findings. Microfilariae were detected by microscopy in the peripheral blood. The patient-side antigen detection test was positive for heartworm antigen, and the ELISA was positive for *D. immitis* antigen. The owner refused further diagnostics and specific treatment. The treatment plan included a 4-week course of doxycycline, praziquantel/milbemycin oxime once per month for 1 year, and restriction of exercise.

### Summary of Cases 1–6

The diagnoses of cases 1–6 were recorded in 2021–2022. All of the dogs were living in the two largest cities in Estonia: Tallinn (59°26’N, 24°45’E) and Tartu (58°22’N, 26°43’E). Four of the dogs were male and two were female; the age range was 2 to 9 years. Three of the dogs had been adopted from an animal shelter; two of the dogs had been imported, one from Croatia and one from Russia; and four of the dogs had no history of import or travel outside of Estonia.

The patient-side antigen detection test was positive for heartworm antigen in all dogs, indicating heartworm disease. Further diagnostic approaches included haematology (cases 1–6), radiology (cases 1–5), serology (cases 1–4, 6) and echocardiographic examination (cases 1–5). Haematology and radiography had been mainly performed by referring veterinarians and were repeated if considered necessary. The final diagnosis of *D. immitis* infection or heartworm disease for all the dogs was made by a board-certified veterinary cardiologist (PFM). According to AHS guidelines [33], *D. immitis* infection or heartworm disease can be confirmed through the identification of circulating microfilariae, by a positive result obtained utilising a different type of antigen test or by ultrasonographic visualisation of adult heartworms within the heart or pulmonary arteries. The results recorded in the patient records supported confirmation of all diagnoses. Microfilariae were detected by microscopy in a peripheral blood sample in one dog (case 6). Serology by ELISA to detect *D. immitis* antigen was positive in five of the dogs (cases 1–4, 6). In two of the dogs (cases 2, 5), filamentous or linear structures consistent with mature parasites were detected in the right ventricle and in the pulmonary arteries in the echocardiographic examination. Abnormal radiographic findings were detected in two dogs (cases 2, 5). Caval syndrome was not detected in any of the dogs. Table 1 summarises the key test results that were used to confirm the diagnoses.

**Table 1:** Six dogs with confirmed diagnosis of *Dirofilaria immitis* infection or heartworm disease, Estonia, 2021–2022

Cases	History of import or travel	Sex	Age (years)	Weight (kg)	Indoor or outdoor dog	Patient-side antigen detection test result	ELISA for detection of *D. immitis* antigen	Echocardiography: adult heartworms	Microscopy: microfilariae
Case 1	None	Male, neutered	5	7.7	Indoor	Positive	Positive		
Case 2	Imported from Croatia	Female, neutered	6	28.7	Outdoor	Positive	Positive	Positive	
Case 3	None	Male	2	19.3	Indoor	Positive	Positive		
Case 4	Imported from Russia	Male, neutered	2	34.2	Indoor	Positive	Positive		
Case 5	None	Female, neutered	5	3.0	Indoor	Positive		Positive	
Case 6	None	Male	9	34.0	Outdoor	Positive	Positive		Positive

Table presents signalment and the key positive test results that allowed the diagnosis to be confirmed in each of the dogs

*ELISA* Enzyme-linked immunosorbent assay

All of the dogs were treated, and based on the records available all of them tolerated the treatment without major complications. The dose, route of administration and timing and duration of each medication included in the treatment plans followed the AHS guidelines [33]. Strict exercise restriction was part of the treatment plan of all the dogs, and their owners were informed about potential complications. Two dogs (cases 3, 4) gained weight, likely due to the exercise restriction. Five dogs (cases 1–5) tested antigen-negative 9 months after the last melarsomine dihydrochloride injection. One dog (case 6) was lost to follow-up.

## Discussion

*Dirofilaria immitis* has spread north and has now been diagnosed in dogs in Estonia. This is important information for veterinarians working in the country and region, as well as for medical doctors and public health professionals because the parasite is zoonotic.

The present study was retrospective and based on information in the patient records of the six dogs included in the study. Information on the total number of canine patients visiting the clinic during the study period or on the total number of the various diagnostic tests performed was not available. Thus, we were unable to estimate the incidence of *D. immitis* infection within the respective subpopulations. A searchable database containing all laboratory results, also negative results, would be useful.

Of the six dogs in the study, two had likely become infected before arriving in Estonia as both originated from countries where *D. immitis* and canine heartworm disease have been reported [19, 36] and the time between arrival in Estonia and the diagnosis of *D. immitis* infection was short. The remainder of the diagnosed infections were likely autochthonous, acquired in Estonia, as the dogs had no history of import or travel outside of the country. The locations where the dogs were living are farther north than the northernmost latitude that a decade ago was predicted to be suitable for *D. immitis* [10, 17].

Three of the six dogs, including the two imported dogs, originated from a dog shelter. In Estonia, medium-sized to large dogs in animal shelters are almost exclusively kept outdoors, which increases the risk of contact with mosquitoes compared to dogs kept indoors. A positive correlation between higher infection rate of *Dirofilaria* spp. and time spent outside has been demonstrated in several studies [29, 37].

The period suitable for the transmission of *D. immitis* in Estonia is 4 to 5 months annually [38, 39]. Previously reported cases of *D. repens* infection [28], together with the cases of *D. immitis* infection reported in the present study, demonstrate that autochthonous circulation of zoonotic *Dirofilaria* spp. is possible and occurring in the country. Several suitable vector species (e.g., *Culex pipens, Culex torrentium, Anopheles maculipennis)* are present in Estonia [15]. Due to climate changes as well as the movement of animals between countries, further and wider spread of zoonotic *Dirofilaria* spp. can be expected.

*Dirofilaria immitis* is a pathogen that needs to be addressed in a One Health approach. Quick diagnosis of canine *D. immitis* infections and heartworm disease is important from both a veterinary and public health point of view. Timely treatment of affected dogs is crucial to avoid life-threatening disease progression and to stop the provision of microfilariae to vectors. Awareness of the local presence of this zoonotic parasite is important, and cross-sectoral collaborations should be established to discuss, study and monitor the situation.

The specific decisions on which tests were performed were made by the treating veterinarians and varied for the six dogs. For all the dogs, a combination of diagnostic tests was used, including the patient-side antigen detection test. In Estonia, this test is widely used in small animal clinics to detect tick-borne diseases (borreliosis, ehrlichiosis, anaplasmosis); consequently, it is possible that some of the *D. immitis* diagnoses may have been incidental findings. The test is highly specific to *D. immitis* and performs well when compared with other methods (antigen detection by ELISA, necropsy) [40]. Nevertheless, cross-reactions may occur with antigens of other nematodes (e.g., *D. repens, A. vasorum, Spirocerca lupi*) [41]. The earliest positive result can be seen 6 months after infection [33, 42].

Information on previous antiparasitic treatment was not included in the patient records of any of the six dogs. A common recommendation for the prevention of canine heartworm disease in endemic areas is the regular use of macrocyclic lactones [43–45]. Another recommended measure is testing dogs 7 months after the end of the mosquito season [33, 42]. Preventive treatment using macrocyclic lactones should be started in puppies as early as possible, but no later than at 8 months of age [42]. In the endemic areas in southern Europe, year-round preventative treatment is recommended [42], while the general recommendation for endemic areas in Europe is the administration of a monthly preventative treatment or a long-acting injectable preventive treatment during the vector season [45]; additionally, the reduction of exposure to mosquitoes is highlighted [45]. The spread of *D. immitis* calls for reconsideration of local recommendations and practices in the regions where the infection emerges.

The goals of the treatment of heartworm disease are to improve the clinical condition of the affected dog and to eliminate all life stages of the parasite with minimal complications. Five of the dogs were treated according to the AHS guidelines [33]. The treatment schedule for infected dogs recommended by AHS includes tetracycline antibiotics (doxycycline), macrocyclic lactones, glucocorticosteroids in certain cases and adulticide treatment with melarsomine dihydrochloride [33]. In two of the dogs, glucocorticosteroids were used at clinician’s discretion. In two dogs, adult heartworms were detected in the right ventricle and in the pulmonary arteries. Caval syndrome (adult heartworms interfering with valve closure, obstruction of the blood flow through the tricuspid valve) was not described in any of the dogs. Surgical extraction of adult worms is recommended in the case of caval syndrome or heavy parasite loads [33, 42]. In all six dogs, strict restriction of exercise was part of the treatment plan. Restricting exercise is the most important measure to minimise the risk of pulmonary thromboembolism and other life-threatening complications [42]. Ensuring the availability of expertise in heartworm disease, its key diagnostic approaches and specific treatment are important in areas where *D. immitis* has recently spread to or is anticipated to spread to.

 Limitations of this work include referral bias, possible recall bias, lack of possibility to confirm the recorded data, limited testing for other pathogens, lack of detailed description of the morphology of the microfilariae and lack of samples for sequencing. However, the diagnoses were confirmed following international guidelines [33].

## Conclusions

The confirmed diagnoses of *D. immitis* infection or heartworm disease in four dogs that had no history of travel or import is an indication of the local circulation of this zoonotic parasite in Estonia and that it has further spread north in Europe than previously described. Microfilariae were observed by microscopy in one of the dogs, which was an outdoor dog, illustrating the likely provision of microfilariae from a reservoir to the vectors. The infection should be included in the list of differential diagnoses in Estonia, also in the absence of a travel history, in both dogs and humans. The availability of diagnostic possibilities and clinical expertise are important. Further epidemiological studies covering the whole country and its dog population are needed, and updating local guidelines for preventive practices should be considered.

## Data Availability

All relevant data are included in the article.
